# Pathophysiology of Barodontalgia: A Case Report and Review of the Literature

**DOI:** 10.1155/2012/453415

**Published:** 2012-12-03

**Authors:** Marcus Stoetzer, Christoph Kuehlhorn, Martin Ruecker, Dirk Ziebolz, Nils Claudius Gellrich, Constantin von See

**Affiliations:** ^1^Department of Oral and Maxillofacial Surgery, Hanover Medical School, Carl-Neuberg-Straße 1, 30625 Hanover, Germany; ^2^Munster Military Medical Center, Munster, Germany; ^3^Department of Preventive Dentistry, Periodontology and Cariology, University of Goettingen Medical Centre, Goettingen, Germany

## Abstract

Changes in ambient pressure occur during flying, diving, or hyperbaric oxygen therapy and can cause different types of pathophysiological conditions and pain including toothache (barodontalgia). We report the case of a patient with severe pain in the region of his mandibular left first molar, which had been satisfactorily restored with a conservative restoration. Pain occurred during an airplane flight and persisted after landing. Radiology revealed a periapical radiolucency in the region of the distal root apex. Pain relief was achieved only after endodontic treatment. On the basis of this paper, we investigated the aetiology and management of barodontalgia. Dentists should advise patients to avoid exposure to pressure changes until all necessary surgical, conservative, and prosthetic procedures have been completed. The influence of pressure divergences should be noted at any time. Under changed environment pressures may be the changing perception of pathologies.

## 1. Introduction

Changes in ambient pressure, for example, during flying, diving or hyperbaric oxygen therapy, can lead to barotrauma. Flying and diving are usually associated with different types of pressure changes. During commercial flights, for example, aircraft personnel are exposed to only minor pressure differences but exposure lasts for a relatively long period of time. By contrast, military and aerobatic pilots are subjected to rapid pressure changes and strong acceleration forces. As a result of the higher density of the surrounding medium, divers are exposed to very high ambient pressures. Compared with aircraft personnel, however, the duration of exposure is usually short. Depending on diving depth and technique, there are considerable differences in the breathing gases used. In addition to changes in ambient pressure, this causes further physiological and metabolic changes in the human body. Barodontalgia was reported to occur during flying at altitudes of 600–1500 m and during diving at depths of 10–25 m [1]. 

A generally accepted classification of barodontalgia was developed by Ferjentsik and Aker [2] and is primarily based on the underlying causes and clinical symptoms (Table1). 

In general, barotrauma is defined as pressure-induced damage that can occur both at high and low pressures. The pathology of barotrauma is directly related to Boyle's law, which states that, if temperature remains constant, the volume of a fixed mass of an ideal gas is inversely proportional to the pressure of the gas. As pressure increases, the volume of a confined gas decreases. Vice versa, volume increases as pressure decreases. Pain during ascent can indicate the presence of a disease of vital pulp tissue (pulpitis). Pain during descent can be indicative of pulp necrosis or facial barotraumas [1].

Pressure differences occur in the human body when a gas-filled cavity cannot communicate with the exterior and pressure cannot be equalized. Clinically, the resulting pressure difference between the gas-filled cavity and the exterior environment can lead to pain, oedema, or vascular gas embolism [3]. This type of pain often occurs in the lungs, the middle ear, or the maxillary sinus (barosinusitis) [4, 5]. In the majority of cases, barosinusitis develops in the presence of acute or chronic maxillary sinusitis. Headaches, numbness, or dental pain in the region of the maxillary posterior teeth occurs as a result of a difference in pressure. A problem in clinical diagnosis is that it is difficult to differentiate between barosinusitis and barodontalgia on the basis of maxillary pain [6, 7]. 

In general, barodontalgia is defined as pain that occurs in the region of the teeth after pressure changes [8]. This phenomenon was first observed in air crews during World War II and was given the name “aerodontalgia.” Later it was also detected in divers. The incidence of this type of tooth pain is 0.26–2.8% in aircraft personnel, air passengers, and divers [6, 9, 10]. A statistical difference between diving and flying personnel has not been reported. 

## 2. Diagnosis and Treatment

A 26-year-old male patient presented with pain in the left mandibular region which had persisted for five days. He reported that the pain had occurred when he had been flying in an airplane and had appeared suddenly at the end of the climb. When asked to describe the intensity of pain using a 0 (no pain) to 10 (worst pain) numerical rating scale (NRS), he rated his pain as 8. At ground level, the patient had been free of pain for approximately five hours. His dental pain had then increased again to a score of 6 to 7 and was described as a dull throbbing local ache. The patient had taken a daily dose of 1600–2400 mg of ibuprofen over a period of four days to relieve his pain since immediate dental care had been unavailable. 

**Figure 1:**
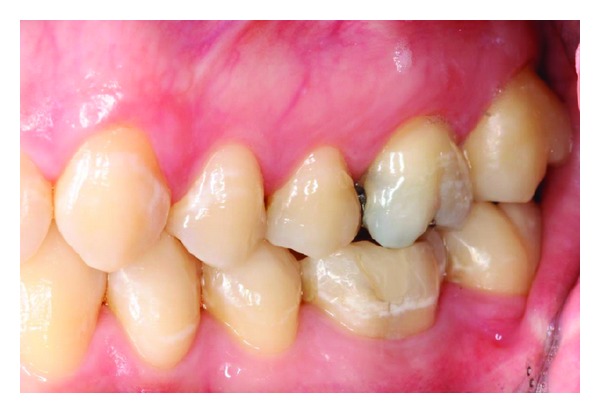
An intraoral clinical examination of the mandible did not reveal macroscopically detectable pathologies.

Upon clinical examination, the mandibular left first molar (tooth 36) (Figure 1), which had been satisfactorily restored with a conservative restoration, was tender to percussion. A periodontal examination of the left mandible was unremarkable. A vitality test of the mandibular left first molar indicated that the tooth was nonvital.

**Figure 2:**
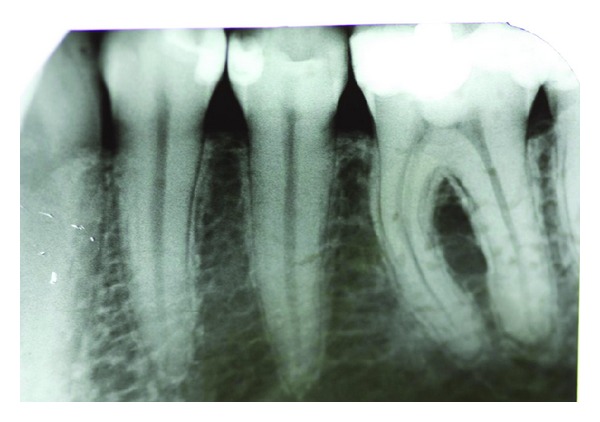
Radiolucency image of tooth 36 showing a periapical radiolucency in the region of the mesial and distal root apices.

A radiographic examination showed a well-restored dentition in the left mandible. A single-tooth radiograph of tooth 36 revealed an enlarged periodontal space and a periapical radiolucency in the region of the distal root of tooth 36 (Figure 2). 

**Figure 3:**
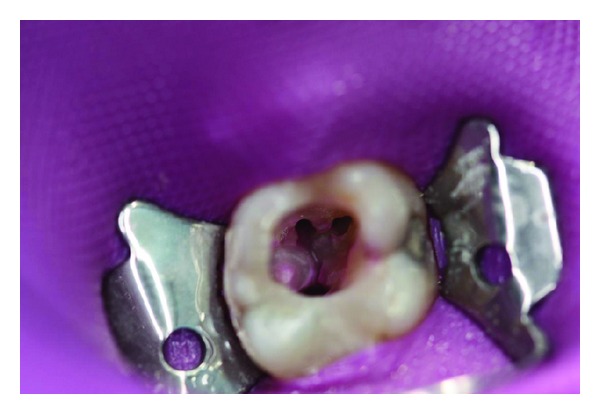
Root canal orifices after enlargement during endodontic treatment.

Following the administration of local anaesthesia and the application of a dental rubber dam, endodontic treatment of tooth 36 was instituted. After access cavity preparation, pulp was removed and three root canals were exposed (Figure 3). During the procedure, no pus was present. Severe bleeding from both mesial root canal orifices, however, was noted. The distal root canal showed in particular gangrenous decomposition of pulp tissue.

The root canals were enlarged using Gates Glidden drills (Dentsply, USA) and prepared with nickel-titanium (Ni-Ti) files according to a standard protocol (Mtwo, VDW, Munich, Germany) with a view to obtaining good visibility of the site. A Ledermix dressing (Haupt Pharma, Wolfratshausen, Germany) was created, and the tooth was temporarily sealed with cement (Havard-Zement, Hofmann & Richter, Berlin, Germany). The presence or absence of cracks was assessed optically using magnifying glasses and a blue-light lamp before and after staining (Mira-2-Ton, Hager and Werken, Duisburg, Germany). No fractures were detected. After the root canal filling was completed, the course of treatment was unremarkable. 

## 3. Discussion

Since the aetiology of barodontalgia is still not completely understood, current dental treatment recommendations for flying and diving personnel are often based on statistical data [11]. A further problem is that barodontalgia can occur irrespective of the type of pressure change, that is, during an increase or a decrease in pressure, and can persist even after pressure equalisation [12]. Possible causative or contributing factors, which, however, are a matter of some controversy, include dentogenic infections, sinusitis, differences in the expansion behaviour of dental enamel and pulp, and pressure-induced movement of fluids from exposed dentine to the pulp [7, 13, 14]. 

In animal experiments, Carlson et al. [13] showed that fluid moved from the dentine to the pulp chamber after cavity preparation in the enamel under hyperbaric conditions. Retrospective studies showed that most patients with clinically manifest barodontalgia had carious lesions or defective restorations extending into the dentine [15]. The clinical implication of this finding is that patients who have carious lesions or have undergone dental treatment including the exposure of dentine, for example, during prosthetic tooth preparation should avoid exposure to pressure changes until definitive treatment. 

In the literature, pulpitis with periapical inflammation or after dental restoration is reported to be the most common cause of barodontalgia. In the case presented here, pain occurred in a tooth with pulp necrosis. The patient was asymptomatic until he was exposed to pressure changes during a flight. In the hypobaric environment, pain developed and persisted even when he was no longer subjected to pressure changes. The pathophysiology of this pain is still not completely understood. It is likely that the clinical symptoms are a result of impaired microcirculation of the pulp.

Apart from dental pain, pressure changes are reported to cause fractures of teeth and dental restorations in pilots and divers [16, 17]. In the case presented here, however, the patient had no such fractures. Gas that is trapped between a tooth and its restoration was long believed to cause fractures of teeth or restorations and lead to barodontalgia. A closer analysis, however, revealed the presence of secondary caries under the fractured restorations. For this reason, a movement of fluids from carious dentine is today believed to be the cause of pulpitis [17].

Indirect pulp capping is currently not recommended in patients exposed to pressure changes. Variations in pressure are believed to adversely affect the regeneration of pulp tissue. In order to avoid possible complications, endodontic treatment or extraction should therefore be performed in cases in which direct pulp capping would be indicated. 

As a rule, persons should undergo a thorough dental examination before being exposed to pressure changes. Treatment must include the restoration of all carious lesions, the removal of all defective restorations, and the management of inflammation. Vitality testing of all teeth is required for the detection and treatment of asymptomatic pulp necrosis [18, 19]. Dentists should advise patients to avoid exposure to pressure changes until all necessary surgical, conservative, and prosthetic procedures have been completed. 

## Figures and Tables

**Table 1:** Classification of barodontalgia.

Class	Cause	Symptoms
Class I	Irreversible pulpitis	Sharp pain on ascent
Class II	Reversible pulpitis	Dull pain on ascent
Class III	Necrotic pulp	Dull pain on descent
Class IV	Periapical pathology	Severe persistent pain on ascent or descent

## References

[1] Zadik Y (2009). Barodontalgia. *Journal of Endodontics*.

[2] Ferjentsik E, Aker F (1982). Barodontalgia: a system of classification. *Military Medicine*.

[3] Hamilton-Farrell M, Bhattacharyya A (2004). Barotrauma. *Injury*.

[4] Kieser J (1997). Sinus barotrauma presenting as acute dental pain. *South African Medical Journal*.

[5] Kressin J (1999). Aviation medicine problems in otorhinolaryngology. *Zeitschrift Fur Arztliche Fortbildung Und Qualitatssicherung*.

[6] Kollmann W (1993). Incidence and possible causes of dental pain during simulated high altitude flights. *Journal of Endodontics*.

[7] Zadik Y, Chapnik L, Goldstein L (2007). In-flight barodontalgia: analysis of 29 cases in military aircrew. *Aviation Space and Environmental Medicine*.

[8] Holowatyj RE (1996). Barodontalgia among flyers: a review of seven cases. *Journal of the Canadian Dental Association*.

[9] Taylor DM, O’Toole KS, Ryan CM (2003). Experienced scuba divers in Australia and the United States suffer considerable injury and morbidity. *Wilderness and Environmental Medicine*.

[10] Del Mar González Santiago M, Martinez-Sahuquillo Marquez A, Bullón Fernández P (2004). Incidence of barodontalgias and their relation to oral/dental condition in personnel with responsibility in military flight. *Medicina Oral*.

[11] Goethe WHG, Bater H, Laban C (1989). Barodontalgia and barotrauma in the human teeth: findings in navy divers, frogmen, and submariners of the Federal Republic of Germany. *Military Medicine*.

[12] Zadik Y (2009). Aviation dentistry: current concepts and practice. *British Dental Journal*.

[13] Carlson OG, Halverson BA, Triplett RG (1983). Dentin permeability under hyperbaric conditions as a possible cause of barodontalgia. *Undersea Biomedical Research*.

[14] Zadik Y (2006). Barodontalgia due to odontogenic inflammation in the jawbone. *Aviation Space and Environmental Medicine*.

[15] Zadik Y, Einy S, Pokroy R, Dayan YB, Goldstein L (2006). Dental fractures on acute exposure to high altitude. *Aviation Space and Environmental Medicine*.

[16] Gunepin M, Derache F, Audoual T (2010). Fracture of a sound tooth in a pilot under hypobaric conditions. *Aviation Space and Environmental Medicine*.

[17] Calder IM, Ramsey JD (1983). Ondontecrexis-the effects of rapid decompression on restored teeth. *Journal of Dentistry*.

[18] Woodmansey K (2008). Class II barodontalgia: review and report of a case. *General Dentistry*.

[19] Zadik Y (2010). Barodontalgia: what have we learned in the past decade?. *Oral Surgery, Oral Medicine, Oral Pathology, Oral Radiology and Endodontology*.

